# Novel cell sources for bone regeneration

**DOI:** 10.1002/mco2.51

**Published:** 2021-05-04

**Authors:** Chenshuang Li, Zane Mills, Zhong Zheng

**Affiliations:** ^1^ Department of Orthodontics, School of Dental Medicine University of Pennsylvania Philadelphia Pennsylvania USA; ^2^ College of Dentistry University of Oklahoma Oklahoma City Oklahoma USA; ^3^ Division of Growth and Development, School of Dentistry University of California Los Angeles California USA; ^4^ Department of Surgery, David Geffen School of Medicine University of California Los Angeles California USA

**Keywords:** bone regeneration, multipotent stem cells, pluripotent stem cells

## Abstract

A plethora of both acute and chronic conditions, including traumatic, degenerative, malignant, or congenital disorders, commonly induce bone disorders often associated with severe persisting pain and limited mobility. Over 1 million surgical procedures involving bone excision, bone grafting, and fracture repair are performed each year in the U.S. alone, resulting in immense levels of public health challenges and corresponding financial burdens. Unfortunately, the innate self‐healing capacity of bone is often inadequate for larger defects over a critical size. Moreover, as direct transplantation of committed osteoblasts is hindered by deficient cell availability, limited cell spreading, and poor survivability, an urgent need for novel cell sources for bone regeneration is concurrent. Thanks to the development in stem cell biology and cell reprogramming technology, many multipotent and pluripotent cells that manifest promising osteogenic potential are considered the regenerative remedy for bone defects. Considering these cells' investigation is still in its relative infancy, each of them offers their own particular challenges that must be conquered before the large‐scale clinical application.

## Graphical abstract

A plethora of both acute and chronic conditions, including traumatic, degenerative, malignant, and congenital varieties, often play key roles in reducing the quality of life for many people. This is particularly true in the case of critical‐size defects where the innate self‐healing capacity of bone is inadequate for a reunion. To date, a diversity of novel multipotent/pluripotent cell sources are regarded as regenerative medicine, particularly for bone regeneration, in virtue of continued worldwide collaboration. Although their potential is irrefutable, each of the cell sources mentioned has its own drawbacks, which must be entirely understood and overcome before they are released for human clinical application.

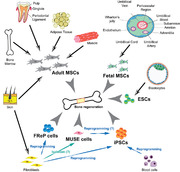

## 1 INTRODUCTION

The typical human skeleton is composed of 206 bones; however, individuals may have varying numbers of bones present, including various small unnamed bones that generally form in high‐friction areas. As the hardest and most rigid structures (aside from the teeth) in the body, bone provides mechanical support, mobility, and load‐bearing capacity. In addition, bones play a critical role in the production of blood cells, the storage of minerals, and the regulation of the endocrine system.

Bone injuries comprise 25‐30% of all musculoskeletal pathologies.^1^ Fortunately, bone is one of the few tissues with a robust innate self‐healing repair mechanism of spontaneous resorption and reformation, allowing small bone injuries to heal in most cases.^2^ However, bone regeneration capacity is not sufficient in regard to reestablishing the skeletal system's integrity and functionality when the damage reaches a critical size, such as those resulting from severe injuries, maxillofacial surgeries of cleft palates, or salvage excision of tumors. A variety of musculoskeletal diseases and congenital conditions also significantly impair bone development and regeneration. In particular, osteoporosis and osteoarthritis are two prevalent diseases within the geriatric population that present major challenges for orthopedic reconstruction. Prevalence of these diseases is shown in the fact that 33‐50% of women and 20‐35% of men over the age of 50 are at risk of experiencing osteoporotic fractures, generating an estimated cost of $25 billion in medical expenses by 2025.^3^, ^4^, ^5^, ^6^, ^7^

Various efforts have been made to improve bone regeneration for decades, including restricted or modified activity, immobilization of injured structure, acupuncture, physical therapy, administration of anti‐inflammatory medication, application of corticosteroids, and revision surgeries. However, these therapies are palliative for bone defect management, as they do not directly stimulate the proliferation, differentiation, and maturation of osteogenic progenitor cells to reestablish the tissue; they merely aleviate the symptoms therein. Due to the advent of tissue engineering and regenerative medicine, new strategies to restore homeostasis in bone deficiency, such as bone graft usage, have been recently proposed.^8^, ^9^, ^10^, ^11^ To date, the autogenous bone graft is still considered the gold standard for reconstructing large skeletal defects, as it not only provides a construction composed of a natural osteoconductive scaffold accompanied by supportive growth factors but also supplies immunotolerated osteogenic‐committed cells.^12^ Although this may be true, the limited resources for autogenous bone grafts and the harvesting procedure's morbidity have fueled the search for alternative bone regeneration approach(s).

In order to be comparable to autogenous bone graft as the ideal blueprint for efficacious bone regeneration, any candidate therapies should provide one or more of the following: (1) a supportive osteoconductive or osteoinductive scaffold, (2) a suitable microenvironment to stimulate cellular osteogenesis differentiation and maturation, and (3) immune‐tolerant osteogenic‐committed cells or progenitor cells harboring the osteogenic potential in vivo.

In regard to the scaffold, functional material design has accelerated the application of biodegradable biomaterials in bone tissue engineering. Several diverse natural proteins (such as collagen,^13^, ^14^, ^15^, ^16^, ^17^ fibrin,^18^, ^19^ and silk^20^, ^21^, ^22^), polysaccharides (such as hyaluronic acid^23^, ^24^, ^25^ and chitosan^26^, ^27^, ^28^, ^29^, ^30^), bioceramics,^31^, ^32^, ^33^ demineralized bone matrix,^34^ synthesized polymers (such as saturated aliphatic polyesters as presented by poly(_D,L_‐lactic acid‐*co*‐glycolic acid),^35^, ^36^ poly[(amino acid ester) phosphazenes]^37^, ^38^), and their composites (such as collagen‐hydroxyapatite‐tricalcium phosphate complex^39^ and polyhydroxyalkanoate/ceramic complex^40^) have been used to construct bone graft substitutes.^41^ Meanwhile, bone graft infection is one of the most serious complications in orthopedic surgery, as they are extremely difficult to treat and typically lead to signficantly worse outcomes.^42^, ^43^, ^44^, ^45^, ^46^, ^47^ Thus, antibiotics, such as gentamicin,^48^ and antiseptics, such as silver nanoparticles,^36^, ^49^, ^50^ are incorporated into the bone grafts to eliminate the contamination/infection and prohibit biofilm formation. These modified scaffolds provide a new avenue for winning the “race” between the infectious organisms that seek to contaminate, colonize, and ultimately infect the graft, and the body's endogenous tissues or embedded exogenous osteogenic progenitors that seek to grow into the graft to attain a functional reunion. Meanwhile, numerous efforts have been devoted to scaffold optimization for accelerating the on growth cells proliferate and differentiate into the mature osseous tissue and deposit bone extracellular matrix (ECM).^51^, ^52^

Multiple growth factors have also been investigated to promote bone formation, among which bone morphogenetic protein 2 (BMP2; Infuse^TM^ Bone Graft) has been approved for use in sinus augmentation and localized alveolar ridge augmentation. Although the osteogenic activity of BMP2 is well documented, its off‐target effects such as postoperative inflammation,^53^, ^54^ osteoclastogenesis,^55^, ^56^ adipogenesis,^54^, ^55^, ^57^, ^58^ and ectopic bone formation^56^ have also been recognized and raised concerns for clinical application of BMP2. Another intensively investigated group factor is BMP7 (also known as osteogenic protein‐1 [OP‐1]). Accumulating evidence demonstrates the efficacy of BMP7 on bone regenration.^59^, ^60^, ^61^, ^62^, ^63^ Unfortunately, the application of BMP2 and BMP7 in the clinical settings faces an array of obstacles, including the high costs, lingering safety concerns (vertebral osteolysis, ectopic bone formation, radiculitis, or cervical soft tissue swelling), considerable failure rates, and controversies. Consequently, recombinant human BMP7 was withdrawn from the market, and restrictions were imposed in the clinical use of recombinant human BMP2.^64^, ^65^, ^66^, ^67^ Aside from the BMP family members, other growth factors also step onto the arena of bone regeneration. For example, in 1999, Ting's group first identified the osteogenic activity of another secretory protein, neural EGFL like 1 (NELL1), in active bone formation sites of human craniosynostosis patients.^68^ Since then, NELL1's osteogenic potency has been verified in several small and large animal models consisting of rodents, sheep, and nonhuman primates^69^, ^70^, ^71^, ^72^ without detecting the adverse effects seen in BMP2 administration.^71^, ^73^, ^74^, ^75^, ^76^ These studies suggest that NELL1 may be an alternative therapeutic option for local or systemic bone regeneration.

In addition to osteogenic growth factors, ultrasound and electrical stimulation are also used to promote bone regeneration.^77^, ^78^ However, a recent systematic review of randomized controlled trials reveals that low‐intensity pulsed ultrasound stimulation did not reduce time to return to work or the number of subsequent operations of patients with fractures, and its benefits on pain management, days to weight‐bearing, and radiographic healing were also to be insignificant.^79^ Although the mechanism is not entirely understood, collagen's piezoelectric property is able to generate a built‐in electric field in the organic bone matrix,^80^ which may activate the membrane receptors on osteoprogenitor cells to subsequently induce osteogenesis.^81^ Beyond this inherent property, faradic products generated around cathodic sites during electrical stimulation also appear to contribute to bone regeneration.^82^ The cations, such as Ca^2+^, can rapidly deposit around the cathode, and anions, such as PO_4_^3−^, HPO_4_^2−^, and OH^−^, subsequently aggregate around the cations.^83^ These depositions result in hydroxyapatite formation at the cathode, which, in turn, promotes bone formation.^83^ In attempts to induce osteogenesis with electric forces, various methods such as direct electrical current,^84^ capacitive coupling,^85^ and inductive coupling,^86^ have been used. Recently, through the engineering of a nanoscale galvanic redox system between silver nanoparticles and 316L stainless steel alloy, novel osteogenic stimulation properties and the bactericidal activities have been introduced into the composite material system,^87^ which offers an innovative strategy to design multifunctional biomaterials for bone formation.

Despite the development in support materials and stimulating methods, cells hold the predominant role in bone regeneration. It is without a doubt that resident stromal resident stem cells, such as perivascular hosted CD146^+^ skeletal stem cells (SSCs)^88^ and newly identified epiphyseal located CD146^−^ SSCs^89^ response to damage on the front line and play essential immunomodulatory and pro‐osteogenic roles in the local environment (*as reviewed* in Refs. 90, 91, 92, 93). Nonetheless, for critical‐size defects, the local osteoprogenitors are insufficient in restoring tissue continuity or function. Several disadvantages, such as the donor site morbidity, inadequate cell availability, poor survivability, restricted proliferation, limited spreading, and dedifferentiation, significantly hinder the clinical use of mature cells, such as osteoblasts.^94^, ^95^, ^96^ Therefore, isolation or generation of safer and more readily available regenerative cell sources remains a major challenge, demanding alternative cell‐based regenerative therapies.

**FIGURE 1:**
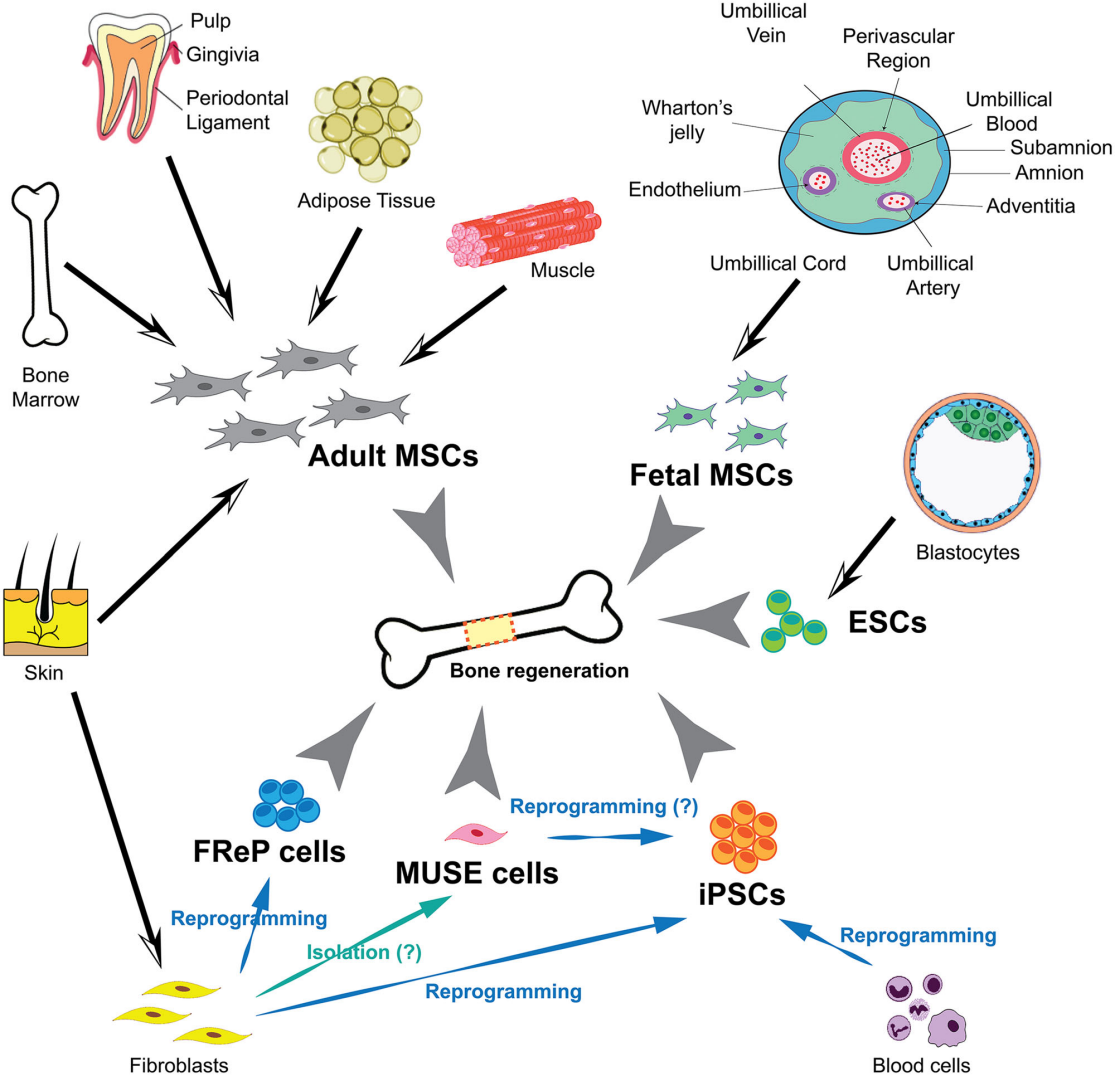
A plethora of both acute and chronic conditions, including traumatic, degenerative, malignant, and congenital varieties, often play key roles in reducing the quality of life for many people. This is particularly true in the case of critical‐size defects where the innate self‐healing capacity of bone is inadequate for a reunion. To date, a diversity of novel multipotent/pluripotent cell sources is regarded as regenerative medicine, particularly for bone regeneration, in virtue of continued worldwide collaboration. Although their potential is irrefutable, each of the cell sources mentioned has its own drawbacks, which must be entirely understood and overcome before they are released for human clinical application

Since Becker, McCulloch, and Till first defined the stem cells functionally in 1963,^97^ stem cells’ pluri‐ or multipotent properties have been a hot investigating topic among the scientific community. This broad excitement has led to continuous improvements in the understanding of stem cell biology, accompanied by worldwide competition for employing stem cell techniques in clinical applications.^98^ This is particularly evident in skeletal regenerative medicine,^99^, ^100^ although inevitable clinical implementation barriers remain intact. A growing diversity of pluri‐ or multipotent cell sources have been investigated for bone regeneration (Figure 1), each of which present unique advantages and, on the other hand, challenges.

## 2 ADULT MESENCHYMAL STEM CELLS (MSCs)

### 2.1 Adult MSCs were first isolated from bone marrow

In 1966, A. J. Friedenstein and colleagues detected the ectopic bone formation by a population of cells isolated from mature mouse bone marrow,^101^ which provided that the first evidence endorsing Cohnheim's hypothesis nonhematopoietic regenerative cells exist in the bone marrow.^102^ Under the appropriate in vitro culture condition, these colony forming unit‐fibroblasts (CFU‐F) can differentiate toward a wide range of cell types, including osteoblasts, chondrocytes, myocytes, and adipocytes. This being the case, these cells were retermed and are now commonly known as bone marrow‐derived stromal cells (BMSCs). On account of their relationship with mesenchymal tissue development and regeneration, BMSCs are recognized as the prototype of MSCs.^103^ Since BMSCs enhanced bone healing in numerous small and large animal models,^104^, ^105^, ^106^, ^107^, ^108^, ^109^ human BMSCs are considered the current gold‐standard cell source for bone regeneration. For example, applying BMSCs to stimulate posterolateral spinal fusion was transferred from the preclinical investigation into the clinical assessment in 2008.^110^ However, the invasive and painful harvesting procedure presents a significant obstacle for BMSCs’ application. Also, the percentage of BMSCs among bone marrow nucleated cells obtained from bone marrow aspiration is typically 0.001‐0.01%.^111^, ^112^ Therefore, large quantities of bone marrow must be procured as starting material to obtain a substantial amount of BMSCs, causing additional donor site morbidity, and thus, remains extremely challenging for BMSCs’ clinical translation. Meanwhile, purification and expansion of BMSCs *via* passaging are generally necessary to eliminate other cell types.^113^ Unfortunately, BMSCs purified by the conventional plastic adherence method and assessed by the fibroblastic morphological criteria are heterogeneous populations containing a diversity of single stem cell‐like and progenitor cells with different lineage commitment.^114^ As a result, even in the seemingly pure preparation of BMSCs, only a subpopulation of BMSCs will be susceptible to osteogenesis.^115^ Further, BMSCs have a relatively low proliferative ability, while the growth factors used to help BMSC expansion can jeopardize their differentiation potential.^116^, ^117^, ^118^

In 2006, Aslan et al isolated a population of CD105 (endoglin)‐expressing cells from bone marrow aspirate.^119^ Since these CD105^+^ cells can be culture‐expanded, express CD90 but not CD14, CD34, CD45, or CD31, and are able to differentiate into osteogenic, chondrogenic, and adipogenic lineages,^119^ they could be considered a subsection of BMSCs according to the Mesenchymal and Tissue Stem Cell Committee of the International Society for Cellular Therapy (ISCT) criteria.^120^ Excitingly, freshly immunoisolated CD105^+^ cells can differentiate into chondrocytes and osteoblasts in vivo with the stimulation with BMP2.^119^ A follow‐up randomized and prospective preliminary study demonstrates the efficacy of these noncultured, immunoisolated CD105^+^ cells on fracture reunion in a clinical setting.^121^ However, these CD105^+^ cells only represent 2.3 ± 0.45% of the mononuclear cells in bone marrow aspirate.^119^ Therefore, novel technologies that easily and safely isolate sufficient highly osteogenesis‐potential BMSCs are still urgently demanded.

### 2.2 Adult MSCs can also be isolated from other tissues

To overcome the aforementioned disadvantages of BMSCs, great efforts have been made to collect MSCs from other tissues. Until now, in addition to bone marrow, MSCs were isolated from a broad range of tissues, such as perichondrium,^122^ cartilage,^123^, ^124^ tendon,^125^ muscle,^126^, ^127^, ^128^ skin,^129^ dental pulp,^130^, ^131^, ^132^ gut,^133^ liver,^134^ and salivary glands.^135^, ^136^ However, isolating MSCs from these tissues also involves invasive and painful harvesting procedures and is limited by the insufficient supply for cell harvesting.

#### 2.2.1 Oral‐derived MSCs

One typical example is the dental pulp. Although studies on a laboratory‐scale demonstrated the benefits of dental‐pulp‐derived MSCs (DMSCs) in the regeneration of craniofacial bone defects,^137^, ^138^, ^139^, ^140^, ^141^, ^142^ the small yield of DMSCs from a single tooth leads to the long‐term cultivation of DMSCs, accompanied with the escalated costs and risks to acquire sufficient DMSCs for clinical implementation. As a result, the regulatory bar for DMSCs seems extremely high. In addition to DMSCs, the oral cavity hosts several other stem cell populations capable of bone regeneration (*as reviewed* in Ref. 92), while similar cell availability limitation and regulatory concerns faced by DMSCs also hindered these cells’ clinical applications.

#### 2.2.2 Adipose‐derived stem cells (ADSCs)

On the other hand, adipose tissue may provide an alternative avenue for MSCs isolation.^143^, ^144^, ^145^, ^146^, ^147^, ^148^ The human adipose tissue is relatively abundant and can generally be obtained through a liposuction procedure.^143^ More importantly, adipose tissue yields a higher amount of MSCs than bone marrow: ADSCs can be harvested at the ratio of 5000 CFU‐F per gram of adipose tissue; in other words, approximately 2% of nucleated cells in processed lipoaspirate are ADSCs.^149^, ^150^ In comparison with BMSCs, ADSCs also have a great proliferation capability^151^, ^152^ and are generally considered stable throughout long‐term in vitro expansion,^153^ exhibiting their potential to be a practical regenerative medicine. Unfortunately, although it has become the second most common cosmetic surgical procedures,^154^, ^155^ lipoaspiration/liposuction is still an invasive and painful surgery performed with anesthesia.^156^, ^157^ Consequently, reports of severely secondary complications of lipoaspiration, such as blood clots, negative reactions to anesthesia, pulmonary complications, infections, and venous thromboembolism, have been significantly increased.^156^, ^158^ Moreover, deaths secondary to lipoaspiration procedures are as high as one death in 5000 surgeries.^155^, ^159^, ^160^, ^161^, ^162^, ^163^, ^164^, ^165^, ^166^, ^167^, ^168^, ^169^, ^170^ Thus, lipoaspiration alone may not be safe for patients with heart problems or blood clotting disorders, women who are pregnant,^156^ or patients with a body mass greater than 35 kg/m^2^ and thus is associated with a very high risk of secondary complications.^155^, ^171^, ^172^, ^173^ More importantly, combined procedures of lipoaspiration for ADSC harvesting and implantation for bone regeneration, particularly with obese or geratiric individuals, will significantly increase the complication rates and often lead to critical safety concerns.^158^

### 2.3 Defination of MSCs is still an open question under investigation

ADSCs are present in a stromal vascular fraction (SVF) that constitutes less than 10% of adipose tissue.^174^, ^175^ SVF is a heterogeneous cell population, including preadipocytes, fibroblasts, vascular smooth muscle cells, endothelial cells, resident monocytes/macrophages, lymphocytes, and putative ADSCs.^174^, ^175^, ^176^, ^177^ Because the presence of mixed stromal and endothelial cells in SVF may dilute and interact with the ADSCs, the benefit of SVF or unpurified ADSCs on bone regeneration is minimal.^178^, ^179^, ^180^ Consequently, a method to purify ADSCs from the SVF is vital, which turns out to post a question in regard to the characterization of MSCs.

Interestingly, MSCs, including BMSCs, DMSCs, ADSCs, and muscle‐derived stem cells,^126^, ^127^, ^128^ are identified in cultures of its dissociated original tissues instead of their native character, frequency, and anatomic location. In 2006, the Mesenchymal and Tissue Stem Cell Committee of the ISCT established a four‐point minimal criterium to define MSCs^120^:

1. Are plastic‐adherent when kept in standard culture conditions?
2. Are phenotypically positive for CD73, CD90, and CD105?
3. Lack of expression of CD45, CD34, CD14 or CD11b, CD19 or CD79α, and human leukocyte antigen‐antigen D related (HLA‐DR).
4. Hold the so‐called “tri‐lineage” differentiation potential towards osteoblasts, adipocytes, and chondroblasts in vitro.

It is worth noticing that CD44, a previously identified essential MSC‐expression cell surface antigen,^181^, ^182^, ^183^ is not admitted to the ISCT standard. These criteria may be suitable for purifying ADSCs from SVF on account of the more homogenous CD9^+^/CD44^+^/CD73^+^/CD90^+^ phenotype presented in the SVF after cultivation.^184^ Nonetheless, accumulating evidence suggests that these criteria may not be completely applicable for identifying ADSCs in vivo or isolating ADSCs from the bulk adipose tissue directly without additional in vitro cultivation steps. For instance, it is known that fibroblasts and stromal cells share the CD73^+^/CD105^+^/CD90^+^/CD44^+^ phenotype, and this quadruple‐positive panel is only sufficient to discriminate these cells from hematopoietic counterparts.^185^, ^186^, ^187^, ^188^

The expression of CD34 is another important issue for characterizing MSCs in vivo. Traditionally, CD34 was considered a unique endothelial and hematopoietic marker, which should not be expressed by MSCs.^189^ Consequently, CD34 expression was used to identify and isolate hematopoietic stem cells (HSCs),^190^ and it was a common misconception that CD34^+^ cells represented the hematopoietic contamination in nonhematopoietic samples. Nevertheless, when Simmons and Torok‐Storb originally generated the monoclonal antibody Stro‐1, which has been extensively used as a selection tool of MSCs, CD34^+^ bone marrow cells were employed as the immunogen.^191^ Surprisingly enough, CD34^+^ cells constitute the majority of SVF cells (60‐80%),^192^, ^193^ and ADSCs harvested from lipoaspirate before in vitro cultivation exhibited some degree of CD34 expression,^194^, ^195^, ^196^, ^197^, ^198^ suggesting that CD34 may not be a real negative marker of MSCs in vivo.^199^, ^200^ Meanwhile, CD34 expression is also present in various stem/progenitor cells, including muscle satellite cells,^201^ hair follicle stem cells,^202^, ^203^ and keratinocyte stem cells.^204^ Further studies show that the standard passaging of ADSCs gradually declines the expression of CD34,^198^ accompanied by the downregulation of other MSC‐associated markers such as CD106, CD146, and CD271.^196^, ^205^, ^206^, ^207^ Be this as it may, the expression of ISTC classified MSC markers CD73, CD90, and CD105 is increased upon cell expansion in vitro.^196^ The similar disappearance of CD34 expression was duplicated in muscle satellite cells when the cells were propagated and differentiated into adipogenic cells.^208^ Additionally, upon activation, the quiescent CD34^+^ keratocytes lost the CD34 expression and acquired a fibroblastic phenotype.^209^, ^210^, ^211^ Further, the high expression level of Stro‐1 antigens in ADSCs is also diminished in response to passaging or induced differentiation.^212^ When considered together, these phenotypical shifts represent a response of multipotent cells to the environmental changes that induce an activation/differentiation from their in vivo quiescent state and indicate that CD34 could be treated as a common marker of quiescent multipotent stem/progenitor population, including ADSCs in vivo.^213^

### 2.4 Adult MSCs are tightly related to pericytes and adventitial cells

Through their emperrical work, Tintut et al demonstrated the multilineage differentiation potency of a subpopulation of vascular cells.^214^ Together with the distribution of Stro‐1 antigens in adipose tissue predominantly located in the endothelium of arterioles, capillaries, and some veins,^215^ this report inspired an enthusiastic investigation to reveal the relationship between MSCs and perivascular cells. In mice, ADSCs have been shown to reside in the adipose vasculature^216^ with the expression of CD34 and stem cell antigen‐1 (Sca‐1; a marker for tissue‐resident stem/progenitor cells^217^) as well as three mura cell markers: α‐smooth muscle actin (α‐SMA), β‐type platelet‐derived growth factor receptor (PDGFRβ), and neural/glial antigen 2 (NG2). In 2008, Péault's group demonstrated that pericytes derived from multiple human organs (including white adipose tissue, skeletal muscle, pancreas, placenta, heart, skin, lung, brain, eye, gut, bone marrow, and umbilical cord) display a CD34^−^/CD45^−^/HLA‐DR^−^/CD44^+^/CD73^+^/CD90^+^/CD105^+^ phenotype after in vitro cultivation.^218^ Moreover, when cultured in suitable conditions, pericytes exhibit the capability for colonial formation as well as osteogenic, chondrogenic, and adipogenic differentiation, which qualified them as MSCs based on the ISCT standard.^218^ This astonishing equivalency of pericytes with MSCs soon after drew the scientific community's attention and led to the hypothesis that “all” MSCs are derived from pericytes.^219^

The comparison between ADSCs and pericytes further emphasizes this theory. Intrestingly, like ADSCs, intact pericytes in their native origin are positive for α‐SMA, PGDFRβ, and NG2 expression, which is not diminished during in vitro expending.^218^ However, unlike mouse ADSCs that are CD34^+^ in vivo and early‐passage in vitro,^198^, ^216^ lack of CD34 expression in pericytes led to confusion regarding their natural affiliation.

In addition to the pericytes that closely associate with microvessel endothelial cells surrounding capillaries and microvessels, multipotent progenitor cells with MSC characters have also been isolated from the bovine artery wall^214^ and the tunica adventitia of the human pulmonary artery,^220^ which suggests the existence of nonpericyte perivascular cells as alternative originators of MSCs.^221^ After exclusion of myogenic (CD56) and hematopoietic (CD45) populations, two distinct populations derived from human white adipose tissue without the expression of endothelium‐specific antigen CD31 could give rise to MSCs: CD146^+^/CD34^−^ pericytes and a second CD146^−^/CD34^+^ population,^222^ because the clones developed from the CD146^−^/CD34^+^ cells displayed the MSC hallmark molecules CD44, CD73, CD90, and CD105, and can undergo osteogenic, adipogenic, and chondrogenic differentiation.^222^ Besides, flow cytometry analysis similarly confirmed that these CD146^−^/CD34^+^ adventitial cells homogeneously express the typical MSC‐associated markers CD44, CD73, CD90, and CD105 in their native origin.^222^ Compared with pericytes, adventitial cells surround the largest vessels and are not closely associated with endothelial cells.^222^, ^223^ Because adventitial cells hold the capability to differentiate into pericyte‐like cells under inductive conditions in vitro, they are proposed to be the precursors of pericytes.^222^, ^223^ A study evaluated human lipoaspirate from 70 donors to reveal that pericytes and adventitial cells comprise an average of 17.1% and 22.5% of SVF, respectively, which, in turn, accounts for approximately 39.6% of total nonadipocyte lipoaspirate cells or 3.96% of total adipose‐nucleated cells.^178^

Although distinguishing these two populations provides significant impacts for stem cell biology research, combining pericytes and adventitial cells together—collectively termed perivascular stem cells (PSCs)—may maximize the MSC source in a clinical setting, especially when autologous cells are used to avoid immunogenic rejection. To bypass the in vitro cultivation steps for the plastic‐adherent cells, which generally takes weeks and increases the risk of spontaneous cellular transformation, Péault's group established a simple sorting procedure to safely and effectively obtain PSCs from routine liposuction.^224^ In this procedure, adipose tissues obtained from liposuction or lipectomy are first digested with collagenase and centrifuged to remove adipocytes. Then, the CD31^−^/CD45^−^/CD34^−^/CD146^+^ pericytes and CD31^−^/CD45^−^/CD34^+^/CD146^−^ adventitial cells are collected from the yielded SVF by fluorescent‐activated cell sorting (FACS).^178^ Thanks to the effects of a multidisciplinary research group led by Drs. Péault and Soo, the entire procedure has been optimized to be completed in a few hours, making the direct sorting‐based PSC application feasible in a clinical setting. As expected, the purified PSCs enhanced bone formation in animal models and were superior to SVF in forming bones.^178^, ^225^, ^226^, ^227^ Thus, purified PSCs seem to be an alternative MSCs source to confer bone formation.

### 2.5 Adult MSCs may benefit tissue repair via bioactive soluble factor production and secreation

In the last few decades, there has been a debate regarding the way in which MSCs ameliorate tissue damages. One possibility is that MSCs directly differentiate or transdifferentiate into parenchymal cells.^228^ Yet, previous studies showed a surprisingly low (less than 1%) and transient engraftment of MSCs in newly formed tissue given the associated therapeutic efficacy.^229^, ^230^ A recent study that tracked the fate of pericytes in vivo in injured skeletal muscle or brain suggested that pericytes did not transdifferentiate as progenitor cells in these two circumstances, further questioning the direct engraftment of MSCs in tissue regeneration in vivo.^231^ Currently, it is believed that the long‐lasting therapeutic benefits of MSCs rely on their bioactive soluble factor production and secretion.^232^, ^233^, ^234^ Particularly, by secreting trophic factors (growth factors, cytokines, and specific proteins), MSCs present their regenerative potency in neurovascular and musculoskeletal therapies.^235^, ^236^ MSCs also produce multiple inflammatory cytokines to modulate the interaction between osteoblast‐lineage and monocyte‐macrophage‐osteoclast lineage; both of which are essential for bone remodeling.^2^, ^237^ Viewing MSCs as “an injury drugstore,”^176^, ^232^, ^238^, ^239^, ^240^ Dr. Arnorld I. Caplan, a pioneer of MSCs research,^103^ suggested renaming the MSCs as “the Medicinal Signaling Cells” to more accurately depict their function in nature.^232^, ^234^, ^241^

### 2.6 Application of adult MSCs in tissue regeneration faces multiple obstacles

#### 2.6.1 Risk of rejection

Similarly to the way in which autologous cell source is the best choice for clinical application, allogeneic MSCs must be considered in some scenarios such as the geriatric population, who are the primary targets for stem cell therapy since the therapeutic effectiveness of MSCs is dependent upon the age of the donor.^242^, ^243^, ^244^, ^245^ MSCs were previously considered immunoprivileged^246^ because undifferentiated MSCs express low to intermediate levels of HLA class I and negligible to low HLA class II.^120^, ^247^ However, MSCs exposed to interferon (IFN)‐γ or differentiated into mature cell types can significantly express more HLA class I and HLA class II.^248^, ^249^ Besides, long‐term in vitro culture was reported to impair the immunosuppressive activity of MSCs.^250^ Moreover, animal model studies displayed a trend of the early death of allogeneic MSCs,^251^, ^252^, ^253^ confirming in the human autopsy of patients who received allogeneic MSCs within a year.^254^ Furthermore, rejection and chronic immune responses of allogeneic MSCs have also been reported in animal studies and human clinical trials.^255^, ^256^, ^257^ Therefore, the current view of MSCs is “immunoevasive” instead of “immune‐privileged.”^258^, ^259^ Accurately measuring immune responses following MSCs treatment in a timely manner is necessary to assess the safety of allogeneic MSCs application. Also, developing novel technologies to prolong the “escaping” status of allogeneic MSCs from the donor's immune system may prolong their persistence in vivo and improve their clinical outcomes.

#### 2.6.2 Risk of tumor formation

Another prudence of MSCs’ usage is their association with tumorigenesis. In 2005, Rubio et al reported the malignant transformation of human ADSCs that had passaged more than 4 months in vitro.^260^ In the same year, Wang et al identified an outgrowth transformed subpopulation of cultured human BMSCs with a round, cuboidal to short spindle shape, distinguished from the typical MSCs.^261^ These cells later termed transformed mesenchymal cells (TMCs), also displayed contact‐independent growth and anchorage‐independent growth when released into the suspension^261^—typical phenotypes seen in tumorigenic cells with metastatic potential.^262^ Rosland et al reported that the ratio of spontaneous malignant transformation of human BMSCs to be as high as 45.8%.^263^ TMCs were also obtained in cultured mouse and monkey BMSCs,^264^, ^265^ while injection of mouse and monkey TMCs resulted in tumor formation in recipient animals.^265^, ^266^ Follow‐up studies argued that spontaneous transformation of MSCs might be false, and the so‐called TMCs may arise from the cross‐contamination with malignant cells that were residents in origin, such as fibrosarcoma and osteosarcoma.^267^, ^268^, ^269^ This claim, whether correct or incorrect, highly emphasizes the drawback of the MSC expansion procedure. Nevertheless, this explanation may not be sufficient for lowering the tumorigenic caution for in vivo MSC implementation, especially for bone regeneration, as bone provides one of the most congenial metastatic microenvironment for tumor progression.^270^, ^271^ Recent studies have shown that purified human MSCs developed chromosomal aberrations during cultivation^272^ and underwent spontaneous tumorigenic transformation,^273^ which cannot be explained by the cross‐contamination theory. Recently, a significant amount of efforts have been devoted to optimizing the cultivation of MSCs,^274^, ^275^ which may eventually result in a practical strategy to control the spontaneous tumorigenic transformation of MSCs.

Certainly, accumulating data have also clearly demonstrated the unbalanced signal transduction in MSCs directly lead to sarcoma formation in vivo.^276^ Besides, the immune suppression potential of MSCs may diminish T‐cell proliferation, thus weakening the antineoplastic response.^277^, ^278^, ^279^, ^280^, ^281^, ^282^, ^283^, ^284^ More importantly, various cytokine, chemokines, and growth factors secreted by MSCs have been shown to increase the proliferation, migration, and angiogenesis of tumor cells.^276^ Akimoto et al revealed that ADMCs promote the growth of cocultured glioblastoma multiforme (GBM) cells in vitro and support GBM development in vivo by at least two distinct mechanisms‐enhancing angiogenesis and inhibiting apoptosis.^285^ Meanwhile, Ren et al reported that similar to tumor‐derived MSCs, tumor necrosis factor α (TNFα)‐pretreated BMSCs enhanced tumor progression by recruiting more macrophages into tumor.^286^ This discovery highlights the possibility that, with an inflammatory stimulation, normal MSCs can convert into a more tumor‐promising phenotype usually found in the tumor microenvironment. These direct and/or indirect involvements of MSCs in tumorigenesis suggest that MSCs hold a high risk for bone reconstruction patients with a history of malignancies.

Taken together, as the current gold standard cell source for bone engineering therapies, adult MSCs from different tissue were intensively investigated (Table 1). The identity, function, safety, and efficacy of these cells are still debatable. Aiming to answer these questions and optimize the clinical application of adult MSCs, a large‐scale, expensive, and time‐consuming investigation is inevitable, which will require a global collaboration of academia and pharmaceutical companies. In addition, the regulation of MSC‐based therapies must be fully and accurately implemented, and most importantly, the long‐term follow‐ups that define the associated risks must be carried out in the clinical trials.

**TABLE 1:** Comparison among adult MSCs derived from different tissues

Cell types	Pros	Cons
BMSCs	Prototype of Adult MSCs Current gold‐standard for stem‐cell based therapies	Relatively low yield Purification and expansion generally needed Heterogenous Relatively low proliferative capability
Oral‐derived MSCs (including DMSCs)	Less invasive harvesting procedure	Low yield Long‐term cultivation needed
ADSCs	Relatively high proliferation capability Relatively stable through long‐term in vitro expansion No‐cultivation protocol has been established	Associated with severe secondary complications

## 3 FETAL MSCs

In addition to adult MSCs mentioned above, less mature MSCs isolated from the umbilical cord have been considered for bone regeneration. These umbilical cord‐derived MSCs (UCMSCs) have similar surface marker expression, high differentiation potential, and low immunogenicity compared with BMSCs.^287^, ^288^, ^289^, ^290^, ^291^, ^292^ Likewise, UCMSCs and BMSCs share the mitogen‐activated protein kinase (MAPK) signal pathway for osteogenic commitment and differentiation.^293^, ^294^ UCMSCs are isolated from umbilical cords, a generally discarded tissue, without ethical concerns,^295^, ^296^, ^297^ potential pain, and medical or surgical risks such as bleeding and anesthetic adminstration.^298^ Moreover, in comparison with adult MSCs, UCMSCs share a high expansion capability with other fetal‐derived stem cells^183^, ^299^ but rarely transform into tumor‐associated fibroblasts.^300^ These advantages support the potential of UCMSCs for bone regeneration.^301^

The human umbilical cord consists of an outer amniotic membrane (amniotic epithelium) that envelops a mucoid connective tissue, which can be characterized as three regions lacking clearly visible structural boundaries: subamnion, Wharton's jelly, and adventitia (a strong, elastic muscle‐like tissue layer), along with three blood vessels (a vein and two arteries).^302^ Among them, Wharton's jelly contains the most abundant source of UCMSCs.^303^ A population of plastic‐adherence, spindle‐shaped cells that express CD44, CD73, CD90, and CD105, but not the hematopoietic markers CD14, CD34, or CD35, can also be isolated from the amniotic membrane,^304^, ^305^, ^306^ although the amniotic membrane was previously considered as solely epithelium and not a source of MSCs.^307^ These amniotic membrane‐derived cells also have the tri‐lineage differentiation potential and can be recognized by Stro‐1. Thus, according to ISCT, they are qualified as MSCs and termed as amniotic membrane‐derived MSCs (AMMSCs). A recent study showed that the proliferation and self‐renewal capacity of AMMSCs are significantly lower than Wharton's jelly‐derived UCMSCs,^308^ indicating the disadvantages of AMMSCs to fulfill the clinical scale requirement in comparison with UCMSCs. Although recent comparison studies suggest that Wharton's jelly‐derived UCMSCs offer the best clinical utility (mainly due to the high isolation percentage and less nonstem cell contaminants to avoid excessive in vitro purification and expansion^309^), the time‐consuming and labor‐intensive dissection of the cord into discrete regions may not be necessary to obtain a valuable population of cells for clinical application,^310^ particularly when FACS‐based purification is employed.

Kargozer et al revealed that implantation of human UCMSCs with three‐dimensional bioactive glass/gelatin scaffold in critical‐sized calvarial defects resulted in a similar degree of bone formation compared to those implanted with unpurified human ADSCs, which is statistically lower than that of the human BMSC‐implanted group.^179^ Interestingly, neovascularization was significantly increased in the human UCMSC group, leading to better healing than using the unpurified human ADSCs.^179^ In another study using RGD (Arg‐Gly‐Asp)‐peptide‐coated macroporous tetracalcium phosphate and dicalcium phosphate anhydrous as a scaffold, human BMSCs and UCMSCs resulted in similar levels of bone formation and vascularization in critical‐sized calvarial defects.^311^ In addition to the influences of the different scaffold materials, another explanation of these paradoxical results could be the known fact that the yield and the differentiation potential of USMSCs highly depend on the method of cell isolation.^292^, ^294^, ^312^ Therefore, a standard isolation/purification methodology should be established and validated before the clinical application of UCMSCs.

Although UCMSCs hold the ability to modulate natural killer (NK) cells and promote regulatory T (T_reg_) cell expansion and thus present a lower rejection risk,^313^, ^314^ immunogenic concern remains the main obstacle for their allogeneic usage.^311^ Meanwhile, the proper cryopreservation of the umbilical cord from childbirth for an extended time is an essential step for autologous usage, which is accompanied by nonnegligible financial and resource inputs.

Umbilical cord blood (UCB) was also recognized as a source of MSCs,^315^, ^316^ although UCB has been considered a reliable source of HSCs for a long time.^317^ These UCB‐derived MSCs (UCBMSCs) were also explored as an alternative cell source for bone tissue engineering and regeneration.^318^, ^319^ Contrary to ADSCs that support GBM development, UCBMSCs inhibit GBM growth while simultaneously inducing apoptosis,^285^ suggesting that UCBMSCs are much safer than adult MSCs for clinical usage. However, UCBMSCs shared a similar immunogenic concern for allogeneic usage and costly storage difficulty for autologous application with UCMSCs. In addition, the yield of MSCs from UCB is extremely low, and the isolation of UCBMSCs is not guaranteed as UCMSCs, while UCMSCs also exhibit a great advantage in terms of proliferation.^320^, ^321^, ^322^ Amniotic fluid is another potential MSCs source^323^, ^324^, ^325^; however, due to the invasive procedure, limited availability, and ethical concern, the yielded amniotic fluid MSCs (AFMSCs) will not be further discussed in this chapter, as there are no superior clinical benefits to use AFMSCs than to use aforementioned fetal MSCs based on the current understanding.

Generally, using fetal MSCs for tissue regeneration seems to be associated with higher technological and regulatory standards, as well as a significant financial burden. Making the fetal MSC‐based therapies to be standard care available for everyone is tremendously challenging in reality, although it holds remarkable scientific interest and may be practical for some specific circumstances.

## 4 EMBRYONIC STEM CELLS (ESCs)

In 1998, Thomson et al first derived the human ESCs from blastocysts.^326^ Briefly, the inner cell mass from the blastocyst stage of embryos is separated from the trophectoderm and plated onto mouse embryonic fibroblast feeder cells to form human ESC colonies.^326^ These human ESCs have normal karyotypes, exhibit high levels of telomerase activity, and display prolonged undifferentiated proliferation. They also express cell surface markers that characterize primate ESCs, present the ability to generate embryoid body (EB) in vitro, maintain the developmental potential to form trophoblast and derivatives of all three embryonic germ layers, and produce teratomas after injection into severe combined immunodeficient (SCID)‐beige mice.

Since their discovery, human ESCs have been broadly used for drug discovery and development.^327^, ^328^, ^329^, ^330^, ^331^ As holding the pluripotent differentiation potential to any tissues, human ESCs have also been investigated as regenerative medicine, including the osteogenic aspect.^332^, ^333^, ^334^, ^335^, ^336^ Human ESC‐based therapies develop very quickly, ie, from Thomson's discovery of human ESCs to their clinical trial for spinal cord injury repair in 2009 (ClinicalTrials.gov Identifier: NCT01217008), it took merely 12 years.

Nevertheless, three inevitable impediments must be surmounted prior to the broad application of ESCs in humans. First, vigorous debates over ESC research and application ethics are continuing, as the human embryos have to be destroyed during the isolation of ESCs.^337^, ^338^ To those who believe that “human life begins at conception” and an embryo is a person with the same moral status as an adult or a live‐born child, taking a blastocyst and removing the inner cell mass to derive an ESC is equivalent to murder.^339^ On the contrary, many others believe that an embryo becomes a person in a moral sense at a later stage of development than fertilization. Taking this into account, the International Society for Stem Cell Research (ISSCR) issued a regulatory guideline that mandates the research on human embryos should be limited to the first two weeks after fertilization.^340^ Although this “14‐day rule” reflects the laws of at least 12 countries,^340^ different administrations may hold varying positions in regard to the creation of new human ESCs, as seen in the Bush administration versus the Obama administration.^341^

Second, the immunogenicity of ESCs should be disclosed, as their clinical usage is clearly allogeneic. To date, reports about the immunological properties of ESCs are still controversial, consisting of those that claim ESCs are uniquely immunoprivileged, those reveal that ESCs hold negligible immunogenicity, and those suggest ESCs can trigger an immune response.^342^, ^343^, ^344^, ^345^, ^346^, ^347^

Last but not least, the genomic instability^348^, ^349^, ^350^, ^351^, ^352^, ^353^ and tumorigenic nature^354^, ^355^, ^356^ of ESCs raise a credible concern for their implantation in human bodies. Therefore, although the investigation of ESCs may pave the path for the investigation of reprogrammed pluripotent or multipotent cells, as discussed below, the clinical usage of ESCs seems impractical in the present stage.

## 5 INDUCED PLURIPOTENT STEM CELLS (iPSCs)

### 5.1 Generation iPSCs from diverse somatic cell origins in vitro starts a new era of cellular biology and regeneration medicine

Since circa 2006, somatic cells are now able to be reprogrammed into an ESC‐like state with pluripotency utilizing viral‐mediated genomic integration of a panel of transcriptional factors essential for embryonic development.^357^, ^358^ The potential to use a patient's own cells to create iPSCs provides a promising new venue for personalized cell therapies by overcoming the ethical paradox and potential immunogenicity of ESCs and fetal MSCs mentioned above. In addition, iPSCs can be directly derived from easily accessible and expandable dermal fibroblasts^359^ and blood cells,^360^ which places iPSCs above adult MSCs by avoiding the invasive harvest pressures to generate sufficient cell source. In acknowledgement of this breakthrough discovery, Dr. Shinya Yamanaka at Kyoko University was awarded the 2012 Nobel Prize in Physiology or Medicine.

Recently, there has been great enthusiasm for applying iPSCs for bone regeneration, with or without an MSC‐intermediate stage. Initially, creating EBs from iPSCs before MSC generation was involved in gaining iPSC‐derived MSCs (iPSC‐MSCs),^311^, ^361^, ^362^, ^363^, ^364^, ^365^, ^366^, ^367^, ^368^, ^369^, ^370^, ^371^, ^372^, ^373^, ^374^, ^375^, ^376^, ^377^, ^378^, ^379^, ^380^, ^381^, ^382^, ^383^, ^384^, ^385^, ^386^ while soon after, an alternative strategy that obained iPSC‐MSCs from dissociated iPSC colonies without the EB formation step was employed.^387^, ^388^, ^389^, ^390^, ^391^, ^392^, ^393^, ^394^, ^395^, ^396^, ^397^ In comparison with adult MSCs, iPSC‐MSCs not only have the advance in regard to proliferation but also present higher telomerase activity leading to less senescence, which is favorable for clinical application.^385^, ^396^ Meanwhile, directly inducing osteogenic commitment of iPSCs without an MSC intermediate step was also reported by several independent groups around at the same time.^180^, ^386^, ^398^, ^399^, ^400^, ^401^, ^402^, ^403^, ^404^, ^405^

It has been demonstrated that the donor's age has no effects on the differentiation potential of iPSCs,^403^ while some studies have revealed the influence of gender on the epigenetic stability of iPSCs.^406^ From scientific perspective, further investigations are warranted to elucidate the impotence of iPSCs’ epigenetic memory on their osteogenic commitment due to the current conflicting evidence and numerous variables in these studies^369^, ^403^; however, based on the clinical consideration of minimizing the risk of the cell harvesting procedure, dermal fibroblasts may be the top, if not the only, cell choice for iPSC generation.

Due to the fact that complex spatiotemporal signals and molecular interactions regulate the in vivo osteogenic commitment of pluripotent cells, a diversity of stimulation is applied to promote the direct osteogenic differentiation of iPSCs, including electronic stimulation,^379^ chemical inducers,^180^, ^363^, ^365^, ^367^, ^374^, ^375^, ^379^, ^380^, ^382^, ^389^, ^400^, ^402^, ^403^ small molecules,^362^, ^380^, ^404^ growth factors in Refs. 366, 374, 376, 380, 382, 389, 399, 403, gene modification,^361^, ^365^, ^370^, ^377^ as well as modified two‐dimensional^405^ and three‐dimensional microenvironment in Refs. 364, 365, 366, 370, 371, 372, 373, 375, 377, 378, 381, 382, 383, 387, 388, 391, 399, 400, 401, 402, 403. Among which, osteogenic medium containing ascorbic acid, β‐glycerophosphate, and dexamethasone formulation is most commonly used in vitro,^363^, ^364^, ^367^, ^374^, ^375^, ^376^, ^379^, ^382^, ^389^ while the three‐dimensional porous scaffold or hydrogel is popular in vivo. Although some studies suggested that iPSC‐MSCs may not completely differentiate into mature osteoblasts in vitro as evidenced by relatively lower or postponed expression of osteogenic markers, especially those indicating the late‐stage osteogenic development,^375^, ^386^, ^397^, ^398^ the performance of human iPSC‐based therapies on bone regeneration was not worse, and maybe even better, than human BMSCs, ADMCs, and UCMSCs at the same circumstances in vivo.^180^, ^311^, ^375^

### 5.2 Tumorigenesis is a significant drawback for iPSCs’ application in humans

“Above all, do no harm.”^407^ Tumor formation associated with cell transplantation must always be avoided in human use. The widely accepted procedure for iPSC generation, in which transcriptional factors essential for embryonic development (such as Yamanaka factors or Thomson factors) are introduced into the genome of target somatic cells, may induce unwanted gene activation and genomic alterations.^408^ As a result, iPSCs are likely to carry a higher tumorigenicity risk than ESCs.^409^, ^410^, ^411^, ^412^, ^413^ Teratoma formation was confirmed in about 20% of SCID mice that had received osteogenic‐induced iPSC for bone defect regeneration.^367^ iPSCs also possess a potential risk for somatic tumor development, which is not present when using ESCs.^414^ iPSCs’ tumorigenic nature was considered an inevitable subsequence of retroviral or lentiviral transduction, resuling in genetic dysfunction, insertional mutagenesis, and tumor formation with genome integration. Thus, different integration‐free techniques were explored for iPSC generation, including adenovirus,^415^, ^416^ Sendai virus,^417^ expressing plasmid vector,^418^, ^419^, ^420^ episomal vector,^421^, ^422^, ^423^ single mini‐circle vector,^424^ piggyBac transposon‐based vector,^425^ RNA,^426^, ^427^, ^428^, ^429^ and cell‐penetrating protein.^430^, ^431^ However, retroviral‐derived and transgene‐free human iPSCs exhibit similar tumorigenicity with no appreciable difference in teratoma formation capability or teratoma microvascular density.^414^, ^432^ Meanwhile, great efforts are also being devoted to replacing Yamanaka factors (OCT4, SOX2, KLF4, and c‐MYC) or Thomson factors (OCT4, SOX2, NANOG, and LIN28) that tightly associate with tumor progression by defined small molecules to achieve chemical induction of pluripotency (CIP).^433^, ^434^, ^435^, ^436^, ^437^, ^438^, ^439^, ^440^, ^441^, ^442^, ^443^, ^444^, ^445^, ^446^, ^447^ Nonetheless, current research indicates that bromodeoxyuridine (BrdU), a mutation inducer that can incorporate into the newly synthesized DNA by replacing thymidine during DNA replication, is required for CIP.^447^ In addition, iPSCs generated through CIP still possess a tumorigenic nature.^433^, ^434^, ^435^, ^436^, ^437^, ^438^, ^439^, ^440^, ^441^, ^442^, ^443^, ^444^, ^445^, ^446^, ^447^ Some reports suggested that using iPSC‐MSCs may be safer than using iPSCs directly, as MSCs provide a relatively lower risk of tumorigenic deviation;^392^, ^448^ however, the tumor‐supporting potency of MSCs, as mentioned above, will also jeopardize the clinical application of iPSC‐MSCs. In the interim, in order to prevent potential tumor formation from undifferentiated iPSCs, cell purification, such as flow cytometry‐ and magnetic bead‐based sorting, is empolyed before transplantation and after differentiation to ensure that only well‐differentiated cells will be transplanted in a generally adopted approach.^449^ Another strategy is to use iPSCs harboring a *chemical‐inducible suicide* gene such that they will have to self‐destruct when tumors are created.^450^, ^451^ Moreover, resveratrol and irradiation were investigated to prohibit tumor formation from iPSCs and their derivatives during in vivo bone repair,^362^, ^367^ which paves a new avenue to battle the “evil side” of iPSCs. Unfortunately, even only a small portion of undifferentiated iPSC contamination is still sufficient to induce tumor formation.^410^, ^450^, ^451^ Thus, purification and selective induction of cell death of undifferentiated iPSCs are inefficient and inadequate to eliminate the risk of teratoma and malignant tumors upon transplantation.^449^

Taken together, while many challenges still exist before the bench‐to‐bedside translation of iPSC techniques, the high capacity of osteogenic differentiation of iPSCs grants the cautious optimism of iPSCs’ promising potential to become a clinical reality in personalized bone tissue engineering and cell therapy.

## 6 FIBROMODULIN (FMOD)‐REPROGRAMMED CELLS

### 6.1 Initial inspiration of fibromodulin reprogramming

Inspired by the pioneer exploration that transferred a somatic cell nucleus to an oocyte^452^, ^453^, ^454^, ^455^, ^456^, ^457^, ^458^ or fused a somatic cell with an ESC^459^, ^460^ to gain pluripotency, *Xenopus* egg extracts,^461^ fish oocyte extracts,^462^ ESC extracts,^463^ and even carcinoma extracts^463^ are used to successfully obtain induced multipotent stem cells (iMSCs) from somatic cells. From a regulatory aspect, the undefined component of these extracts makes it almost impossible to use these iMSCs in humans. However, these studies strongly support the hypothesis that pluripotent cells’ surrounding microenvironment may play an important role in cell fate determination, including maintaining and/or inducing pluripotency.^464^

As mentioned above, UCMSCs are predominantly harvested from Wharton's jelly, a proteoglycan‐rich connective tissue.^292^, ^319^ Interestingly, like other fetal MSCs,^465^, ^466^ UCMSCs seem to lie between the adult MSCs and ESCs on the development map as they present specific markers of ESCs in addition to those of adult MSCs.^313^, ^467^ These observations provoke a question: can we reprogram connective tissue somatic cells to some degree of multipotent/pluripotent by reestablishing a proteoglycan‐rich microenvironment?

Bi et al reported that FMOD, an ECM proteoglycan broadly distributed in connective tissues, is a critical component for maintaining endogenous stem cell niches by modulating the bioactivities of growth factors.^468^ As a 59‐KD small leucine‐rich proteoglycan (SLRP) member, FMOD contains a central region composed of leucine‐rich repeats, with four keratan sulfate chains flanked by disulfide‐bonded terminal domains.^469^, ^470^, ^471^ Holding high conservation among the mammalian species, FMOD core protein binds to an array of molecules including collagen,^469^, ^472^ transforming growth factor β (TGFβ),^473^ and lysyl oxidase.^474^ Accumulating evidence provides that, in addition to its originally described roles in ECM structural support, FMOD also serves as a key regulator of intracellular signaling cascades that govern multiple biological processes,^470^, ^475^ such as angiogenesis.^476^, ^477^, ^478^ During our long‐time investigation into fetal scarless wound healing, we demonstrated that the single loss of FMOD is adequate to induce scar formation in early‐gestation fetal animals, which normally heal without scarring. On the other hand, exogenous administration of FMOD is sufficient in restoring scarless fetal repair to late‐gestation animals.^479^ This evidence not only highlights the essential role of FMOD plays in scarless fetal wound healing, but we also demonstrate that FMOD reduces scar formation in adult cutaneous wounds by eliciting a fetal‐like phenotype of adult dermal fibroblasts.^480^ These studies suggest the potential of FMOD in cell rejuvenation and maybe even reprogramming.

### 6.2 Generation and characterization of FMOD‐reprogrammed (FReP) cells

In 2012, we first reported a strategy to generate multipotent cells from human dermal fibroblasts by continuously stimulating with recombinant human FMOD under a serum‐free condition.^481^ Through using this technology, dermal fibroblasts isolated from donors of different ages and genders have been successfully reprogrammed into a multipotent stage.^481^, ^482^ The yield dome‐shaped, clustered FReP cells can be easily separated from the surrounding spindle‐shaped, monolayer FReP‐basal cells with a Xeno‐free and enzyme‐free reagent developed for passage of human ESCs and iPSCs.^482^ These FReP cells express the ESC/iPSC markers, such as NANOG, SOX2, SSEA4, TRA‐1‐60, and TRA‐1‐81, while their OCT4 expression is lower than that of iPSCs generated from the traditional viral‐mediated method.^481^ The activation of these essential transcriptional factors for cell reprogramming, accompanied by a specific, biphasic Smad3 phosphorylation, was also validated by multiple methods.^481^ Similar to ESCs and iPSCs, FReP cells can form EBs in suspension culture and are capable of differentiating into neuron (ectoderm derivative), pancreatic lineage (endoderm derivative), and multiple mesoderm derivatives, such as osteoblasts, cardiomyocytes, skeletal myocytes, and adipocytes in vitro.^481^, ^482^, ^483^, ^484^

### 6.3 FReP cells exhibit superior potential for bone regeneration than iPSCs

**FIGURE 2:**
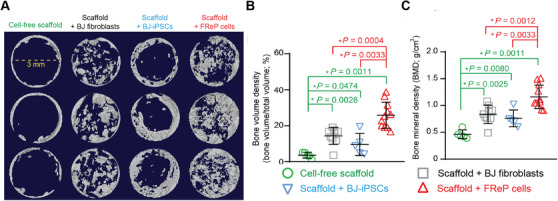
Radiographic analysis of bone regeneration in critical‐sized SCID mouse calvarial defects at week eight postimplantation. Three days prior to implantation, 5 × 10^5^ tested cells were seeded on porous poly(_D,L_‐lactic acid‐*co*‐glycolic acid)/hydroxyapatite scaffold and culture in an osteogenic medium containing ascorbic acid, β‐glycerophosphate, and dexamethasone for in vitro induction. (A) MicroCT images of bone regeneration in critical‐sized calvarial defects implanted with cell‐free scaffold (*N* = 5), scaffold + parental BJ fibroblasts (*N* = 9), scaffold + BJ fibroblast‐derived iPSC through conventional c retrovirus‐mediated method (BJ‐iPSCs; *N* = 5), and scaffold + BJ‐FReP cells (*N* = 11). Images were documented at a resolution of 20.0 μm. Quantification of bone volume density (B) and bone mineral density (C) revealed that implantation of FReP cells resulted in significantly more new bone formation than other groups. *, statistical significance revealed by Mann‐Whitney analysis; green stars indicate the difference from the cell‐free scaffold group; red stars indicate the difference in comparison with the group of scaffold + FReP cells. Modified from Li et al^482^ with permission from Elsevier

**FIGURE 3:**
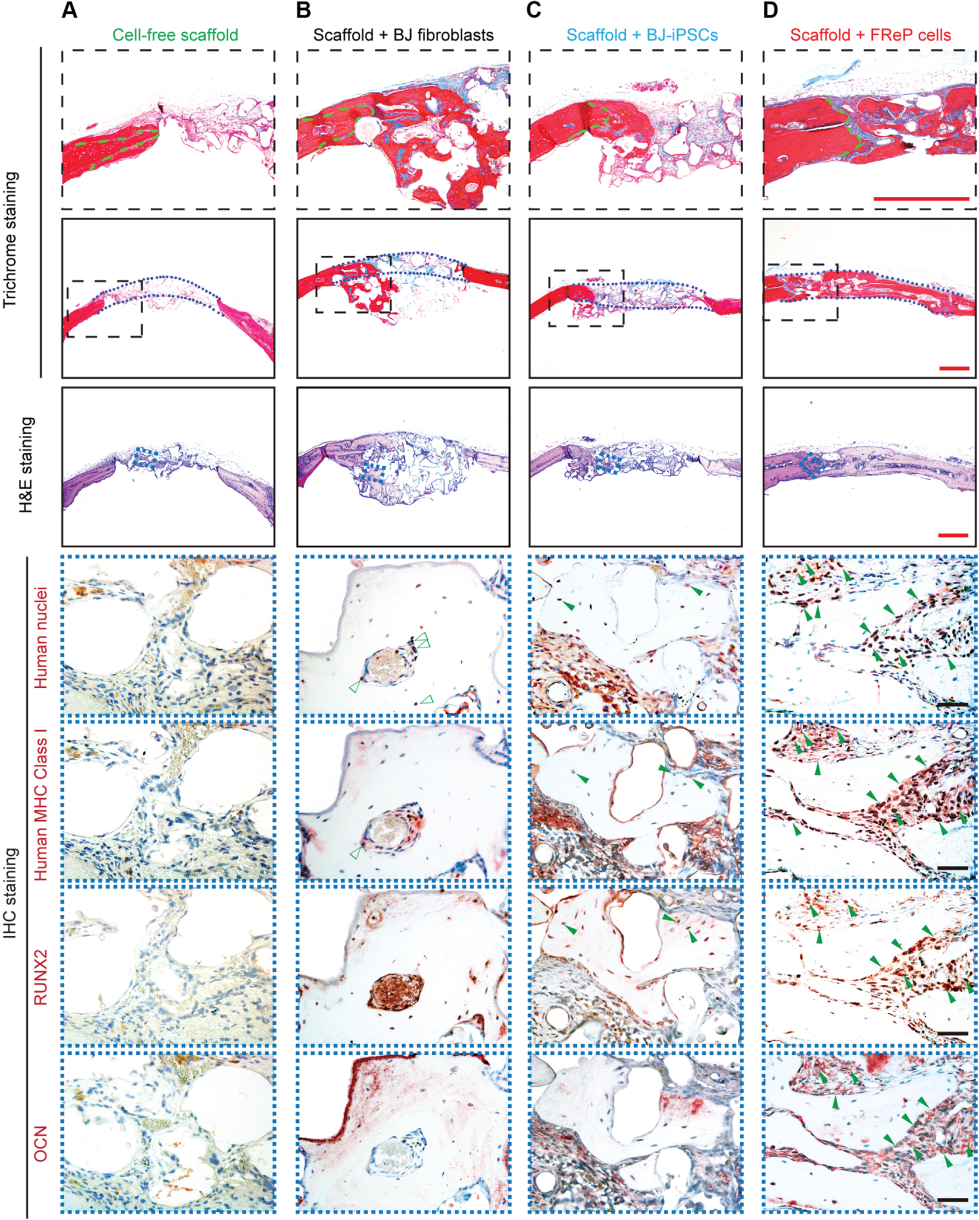
Engraftment, persistence, and osteogenesis of FReP cells in critical‐sized SCID mouse calvarial defects at week eight postimplantation. Hematoxylin and eosin (H&E) and Masson's trichrome staining confirmed that only minimal new bone regeneration occurs in the group implanted with the cell‐free scaffold (A), while implantation of BJ‐fibroblasts resulted in bone formation underneath the calvarial defect with obvious “cyst‐like bone voids” in the newly generated bone area (B). The newly formed bone tissue was predominantly observed at the edge of the defects in the group implanted with iPSCs (C). On the contrary, implantation with FReP cells led to a mineralized bony bridge connecting the two defect ends without ectopic bone formation (D). In Masson Trichrome staining, the mature bone is stained in red, and the osteoid is stained in blue. Green dotted lines outlined the initial edges of the calvarial defects, while blue dotted lines outlined the implantation area, respectively. Furthermore, immunostaining of human nuclei and human major histocompatibility complex (MHC) Class I as well as osteogenic markers runt‐related transcription factor 2 (RUNX2) and osteocalcin (OCN) confirmed the osteogenic differentiation of iPSCs and FReP cells in active osteogenic regions of the defects, while BJ fibroblasts were only detected in the fibrotic area instead of the newly formed bone tissue. Bar = 500 μm (red) or 50 μm (black). Reprinted from Li et al^482^ with permission from Elsevier

Not only did FReP cell exhibit a similar capability of triploblastic differentiation in vitro as ESCs and iPSCs, the in vivo myogenesis and osteogenesis of FReP cells were also documented in SCID mouse models.^481^, ^482^, ^483^, ^484^ Notably, in the broadly accepted critical‐sized calvarial defect model, radiograph analysis revealed significantly more bone formation of the FReP cell implanted group than that of the empty scaffold, parent fibroblasts, and even iPSCs‐implanted groups at eight weeks posttransplantation (Figure 2A).^482^ This observation was further supported by the quantification of bone volume density and bone mineral density (Figure 2B‐2C).^482^ Histological staining also identified a mineralized bony bridge connecting the two defect ends without ectopic bone formation in the FReP cell group. In contrast, the bone formation of the iPSC group was limited at the defect edges.^482^ Additionally, engraftment and differentiation of both iPSCs and FRePs were demonstrated by the colocalization of human markers and osteogenic markers in the newly formed bone,^482^ confirming that both iPSCs and FReP cells are directly involved in the new bone formation in vivo (Figure 3). Considering the significantly higher bone formation correlated with higher density of the FReP cell group when compared to those of the iPSC group,^482^ FReP may be the superior option in bone regeneration efficacy.

### 6.4 FReP cells carry significantly less tumorigenic risk than iPSCs

Like iPSCs, FReP cell generation is unfettered by the ethical and logistical constraints that overshadow the generation of ESCs. Another advantage of FReP cells is that they are generated from a protein‐based technology without genome integration or oncogene activation. Importantly, unlike iPSCs that form teratomas as a consequence of the uncontrolled cellular proliferation,^414^ FReP cells have low proliferative capabilities under undifferentiated circumstances, which can be disrupted by osteogenic or myogenic stimulation.^481^ Under an intramuscular microenvironment, iPSCs implantation led to 25% tumor formation, while no teratoma or other kinds of tumors were generated from FReP cells in SCID mice.^484^ Likewise, when FReP cells were intratesticuarly implanted in Fox Chase SCID‐Beige mouse with Matrigel^®^ carrier, no teratoma was observed in a 4‐month experimental period, while 100% of the animals with iPSC implantation developed teratoma with progressive growth.^484^ Because teratoma formation of FReP cells was not found in the kidney capsule of Fox Chase SCID‐Beige mouse either,^481^ FReP cells are considered to be a safer cell source for regenerative medicine than iPSCs. Albeit, as FReP cells' investigation is still in its infancy, an abundance of in‐depth investigations must be conducted before translating FReP cells to a clinical setting, including but not limited to the optimization of productivity and the long‐time safety and efficacy assessment.

### 6.5 FReP cells and multilineage differentiating stress enduring (MUSE) cells present a group of multipotent cell sources for regenerative medicine

Interestingly, FReP cells bear several critical characteristics of MUSE cells:^481^, ^483^, ^485^ (a) express pluripotent markers, albeit at lower levels than ESCs and iPSCs, (b) hold the capability to differentiate into all three germline cells under specific inductions, (c) have low levels of proto‐oncogenes, such as *LIN28* and *c‐MYC*, (d) retain a stable karyotype, and most importantly, (e) do not form teratomas. Although FReP cells and MUSE cells are both excluded from being considered pluripotent due to the stringent mandatory criteria of teratoma formation when introduced to an in vivo environment, they may represent a different group of cells processing triploblastic differentiation capability that holds tremendous potential in regenerative medicine.

Nevertheless, FReP cell generation is distinct from MUSE cell collection. Activation and isolation of MUSE cells require severe cellular stress conditions, such as lengthy incubation and digestion, hypoxia, and low temperatures,^485^ which assist in killing off all of the other viable cells. FMOD reprogramming does not require hypoxia or low temperatures, and the resultant FReP cells and FReP‐basal cells are both viable. FReP cells resemble quiescent stem cells in multiple ways,^481^ and as such, the mechanism by which FMOD assists in reprogramming demands a thorough exploration. Bearing in mind the multiple striking similarities shared by FReP and MUSE cells, the relationship between these two populations should also be further investigated. MUSE cells are considered a primary source of iPSCs in human fibroblasts in the elite model for iPSC generation.^486^, ^487^ However, the mechanism governing the transition from nontumorigenic MUSE cells to tumorigenic iPSCs remains an enigma. Further exploration into the mechanism of MUSE cell generation, as well as FMOD reprogramming, may also benefit to clarify the molecular roadmap of somatic cell reprogramming in general.

## 7 CONCLUSION

A diversity of novel multipotent/pluripotent cell sources is recruited as regenerative medicine outlets (Figure 1), particularly for bone regeneration in virtue of continued worldwide collaboration. Although their potential is irrefutable, and the opportunity to develop personalized cell therapy (in the cases of iPSCs and FReP cells) is extremely enticing, each of these cell sources has its own obstacles (Table 2) that must be understood entirely and overcome before they may be used on a large scale in a clinical setting. Despite the preliminary efficacy and safety assessment in a laboratory setting, further clinical data are necessary to determine their therapeutic benefits and safety, as well as to optimize their use as a part of the novel regenerative medicine strategy. Furthermore, ways in which we can promote seeding cells survive, growth, and differentiation into desired tissues *via* the implantation vehicle or scaffolds is also an open question for global collaboration, although previous studies may already point out some fundamental directions.^51^, ^52^ We believe that in light of the currently existing evidence, a new era of cell‐based bone regeneration is becoming a reality with the continued collaborative efforts of scientists, physicians, industry, and regulatory agencies.

**TABLE 2:** Summary of novel cell sources for bone regeneration discussed in this review

Cell types	Pros	Cons
Adult MSCs	Intensively investigated Possible benefit of tissue regeneration by secreting trophic factors Immuno‐evasive	Invasive and painful harvesting procedure May not directly differentiate or transdifferentiate into the desired tissues Reported rejection and chronic immune response of allogeneic MSCs Support or directly involved in tumor formation
Fetal MSCs	Isolated from generally discarded tissues without ethical concerns, potential pain, and medical or surgical risks Highly expansible Low rejection risks Rarely transform to tumor‐associated phenotype Induce apoptosis of tumor cells	Lack of standard isolation and purification methodologies Lack of regulatory standards Associated with higher costs
ESCs	Pluripotency Fast development and intensively investigated	Ethical dilemma Data of immunogenicity is not fully revealed Genomic instability Tumorigenic nature
iPSCs	Breakthrough discovery Worldwide collaboration established Could be derived from patients' own cells, such as fibroblasts and blood cells Minimal risk of invasive harvesting procedure and rejection	Genomic instability Signiant tumorigenic risk
FReP cells	Could be derived from patients’ fibroblasts Minimal risk of invasive harvesting procedure and rejection Superior potential for bone regeneration compared to iPSCs No genomic integration or oncogene activation Significantly lower tumorigenic risk	Investigation in its infancy
MUSE cells	No genomic integration or oncogene activation Significantly lower tumorigenic risk	Investigation in its infancy Invasive and painful harvesting procedure Severe cellular stress conditions for purification

## CONFLICT OF INTERESTS

Dr. Zhong Zheng is an inventor on FMOD‐related patents assigned to UCLA, and a founder and an office of Scarless Laboratories Inc., which sublicenses fibromodulin‐related patents from the UC Regents. Dr. Zheng also holds equity in the company.

## CONTRIBUTIONS

Dr. Chenshuang Li drafted the manuscript, Mr. Zane Milles conducted the literature collection and proofreading, and Dr. Zhong Zheng conceived the ideas of the manuscript and provided revisions to the scientific concept. All authors read and approved the final manuscript.

## ETHICS APPROVAL STATEMENT

Ethical approval not applicable to this review article. They will have been obtained from the original authors of the cited references.

## Data Availability

Data sharing not applicable to this article as no new datasets were generated for this review article.
